# Contrasting Food Web Factor and Body Size Relationships with Hg and Se Concentrations in Marine Biota

**DOI:** 10.1371/journal.pone.0074695

**Published:** 2013-09-03

**Authors:** Roxanne Karimi, Michael Frisk, Nicholas S. Fisher

**Affiliations:** School of Marine and Atmospheric Sciences, Stony Brook University, Stony Brook, New York, United States of America; University of Tasmania, Australia

## Abstract

Marine fish and shellfish are primary sources of human exposure to mercury, a potentially toxic metal, and selenium, an essential element that may protect against mercury bioaccumulation and toxicity. Yet we lack a thorough understanding of Hg and Se patterns in common marine taxa, particularly those that are commercially important, and how food web and body size factors differ in their influence on Hg and Se patterns. We compared Hg and Se content among marine fish and invertebrate taxa collected from Long Island, NY, and examined associations between Hg, Se, body length, trophic level (measured by δ^15^N) and degree of pelagic feeding (measured by δ^13^C). Finfish, particularly shark, had high Hg content whereas bivalves generally had high Se content. Both taxonomic differences and variability were larger for Hg than Se, and Hg content explained most of the variation in Hg:Se molar ratios among taxa. Finally, Hg was more strongly associated with length and trophic level across taxa than Se, consistent with a greater degree of Hg bioaccumulation in the body over time, and biomagnification through the food web, respectively. Overall, our findings indicate distinct taxonomic and ecological Hg and Se patterns in commercially important marine biota, and these patterns have nutritional and toxicological implications for seafood-consuming wildlife and humans.

## Introduction

Seafood is an important source of lean protein for humans and piscivorous wildlife. However, the overall nutritional value of individual marine fish and shellfish species is complex and depends on numerous other nutritional factors and contaminants such as mercury. Seafood is a primary source of both mercury (Hg) and selenium (Se) exposure. Mercury is a nonessential, toxic metal that often triggers fish consumption advisories [1], whereas Se is an essential element necessary for growth in phytoplankton [2], animals and humans [3], but also is toxic in high doses [4], [5]. Se can protect against Hg bioaccumulation and toxicity [6]–[8], partly due to the formation of metabolically unavailable Hg-Se compounds [9]. This protective effect can depend on the chemical species present and route of exposure [10]. Yet, there may be a need to incorporate Se into characterizing the risks and benefits of consuming different seafood species [11]. A first step toward this goal is to better understand Hg and Se patterns in common commercial fish and shellfish species, and the factors influencing these patterns.

While many studies have examined Hg (reviewed in [12]) or Se concentrations [13], [14] in aquatic biota separately, few have simultaneously compared Hg and Se concentrations in the same organisms while also examining relationships between these concentrations and environmental factors. Most studies that have measured both Hg and Se content in the same organisms [15]–[23] either focused on organisms that are absent or uncommon in the US seafood diet, or did not directly relate Hg and Se concentrations to environmental factors to improve our understanding of Hg-Se patterns in natural populations. This study addresses these gaps by examining Hg and Se content in multiple, common marine taxa that are commercially important, and relates Hg and Se content to food web and body size factors that are likely to influence Hg-Se patterns in natural populations.

Aquatic organisms obtain both Hg and Se primarily from their diet [24]–[27]. Thus, in addition to the influence of chemical factors on trace element bioavailability (e.g., pH [28], [29], DOC [30]), factors related to diet (e.g., food chain length, trophic level, body size, consumption rate, growth rate) can strongly influence trace element bioaccumulation [31]–[35]. However, few studies have directly compared the role of these factors on Hg and Se concentrations in marine taxa. Such comparisons provide important information for Hg and Se co-exposure and management, such as comparing the extent to which Hg and Se biomagnify, or increase in concentration through the food web. Although biomagnification of Hg, in the organic form of methylmercury, is well-documented [36], [37], evidence for Se biomagnification is inconsistent across studies. For example, estimates of Se trophic transfer factors (ratios of Se concentrations in predator to those in prey) across studies vary over a narrow range encompassing values indicating cases of decreasing Se concentrations with increasing trophic level (trophic transfer factor <1) as well as biomagnification (trophic transfer factor >1) [38], [39]. Experimental evidence also indicates that biomagnification of methylmercury, the dominant form of mercury in fish [40], is likely to be stronger than that of Se in part because loss of assimilated element from the body is lower for methylmercury than selenium [31]. Thus, while the collective evidence suggests stronger biomagnification of Hg than Se, we lack direct comparisons of Hg and Se concentrations in commercially important, marine biota in relation to food web and other ecological and biological factors.

In this study, our primary goals were to compare Se and Hg concentrations in marine fish and invertebrates collected from Long Island marine waters, and examine relationships between these elements and factors commonly thought to influence trace element content, including trophic level [33], [35], [36], importance of habitat-specific food sources in the diet (i.e., degree of pelagic feeding) [41], [42] and body size [43]–[45]. By comparing taxonomic differences and variability of Hg and Se content, our findings help characterize and understand the nutritional and toxicological value of these organisms.

## Methods

### Ethics Statement

All necessary permits were obtained for this field study. New York Department of Environmental Conservation (license # 943, 1272, 1030, and 1633) approved field collection.

### Fish Collection

Fish and shellfish were collected from bays on the north and south shore of Long Island, NY, in accordance to an approved animal protocol at Stony Brook University (IACUC project number 20081587). Fish species, blue crab and squid were collected from three study sites between April and September in 2007 and 2008. Details regarding study sites and sample collection methods are published elsewhere [46]. Briefly, study sites included Port Jefferson Harbor in the Long Island Sound, and the Great South Bay and Shinnecock Bay along the south shore of Long Island. Organisms were collected using otter trawls (all three sites) and beach seines (Port Jefferson Harbor and Shinnecock Bay). In addition, we collected shark muscle tissue samples from the base of shark fins from shark tournaments at Casco Bay, ME (thresher shark, *Alopias vulpinus*), Oak Bluffs, MA (thresher shark), and Montauk, NY (thresher shark and mako shark, *Isurus oxyrinchus*) in August 2010. Sharks from each of these areas are considered to be a part of the same regional population, thus were combined in our data set. Striped bass (*Morone saxatilis*) were collected by seine haul in 2009 from Jamaica Bay on the south shore, and Little Neck and Manhasset Bays on the north shore as part of a separate study conducted by the New York State Department of Conservation [47]. After collection from the field, finfish, shark, crabs and squid were stored frozen until sample preparation. Finally, bivalves were collected by hand from Stony Brook Harbor and Huntington Harbor in the Long Island Sound from August to October 2010. Bivalves were stored in a 20°C refrigerator until dissection.

### Sample Preparation

Frozen fish and invertebrates were thawed before dissection for trace element and stable isotope samples. Prior to dissection, individual, whole-bodied organisms were measured for wet weight and length. Specifically, we measured length as total length for finfish, carapace length for blue crabs, from the end of longest arm to the posterior end of mantle for squid, and shell length for bivalves. Bivalve wet weights included soft tissue only. Weight and length measurements were not available for individual shark specimens. Instead, we used average total length measurements estimated for each shark species collected for this study (mako shark: 122 cm, thresher shark, 152 cm) for all data analyses.

We removed commonly consumed, edible tissues from organisms for trace element (total Hg and Se) analysis. Individual, whole-bodied organisms were rinsed with 0.2 µm filtered Milli-Q water. All tools and surfaces were acid-cleaned using trace metal clean techniques [48]. For finfish trace element samples, using a stainless steel scalpel, we removed axial muscle tissue from underneath the lateral line. When individual fish were small (approximately 6 to 10 cm total length), muscle tissue from multiple individual fish of similar size were composited into a single sample (specifically, bay anchovies (*Anchoa mitchilli*), smaller scup (*Stenotomus chrysops*), and killifish (*Fundulus sp.*). We removed muscle tissue from blue crabs (*Callinectes sapidus*), external mantle tissue and arms for squid (*Loligo pealei*), and muscle tissue at the base of the dorsal fin from sharks. For bivalves, we included all soft tissue from the shell. Trace element sample tissues were added to 15 mL polypropylene metal-free centrifuge tubes and measured for wet weight. We collected sample blanks by rinsing sample preparation tools and surfaces between samples and adding rinse water to blank sample tubes to account for background contamination due to sample preparation.

We collected a parallel set of samples from the same tissues to be measured for C and N stable isotope analyses. Animal tissue samples for stable isotope analyses were placed in Whirlpack bags. All samples were placed in a −80°C freezer overnight and then lyophilized, after which dry weights were recorded. Trace element samples were acid-digested and analyzed for Hg and Se with an inductively coupled plasma mass spectrometer (Agilent 7500cx, Santa Clara, Ca) at the Trace Element Analysis Core Laboratory at Dartmouth College. We measured total mercury content rather than methylmercury primarily because total mercury is more commonly reported in the literature, thus is easier to compare with other studies. Finally, almost all of the mercury in finfish is in the form of methylmercury [40]. Therefore, total mercury is an excellent proxy for methylmercury in these biota. Stable isotope samples were homogenized, weighed in tin capsules and analyzed for isotopic signatures (δ^13^C, ^13^C/^12^C and δ^15^N, ^15^N/^14^N) using a continuous-flow Europa Hydra 20/20 IRMS fit with a Europa ANCA sample combustion unit (Europa Scientific, Cambridge, UK) at the Stable Isotope Facility at UC Davis. Trace element samples were closed vessel microwave digested (MARS Express, CEM Coop, Mathews, NC) with HCl and HNO_3_ (Optima, Fisher Scientific, St Louis, MO).

We digested and analyzed standard reference materials for external quality control of trace metal samples, including NIST SRM mussel tissue 2976, DOLT4 and DORM-3, NRC-CNRC Canada. Additional trace metal quality control procedures included the use of sample duplicates, spike analysis and laboratory blanks. Recoveries between 80 and 120% of the spike amount were accepted to validate the calibration. Gold (200 ppb) was added to in the internal standard mix and the rinse solution to reduce carryover of Hg. Duplicate samples were analyzed after every 10–20 samples. Percent differences between digestion duplicates ranged from approximately <1 to 10%, indicating that Hg carryover did not affect our results. Percent recovery of SRM for Hg were 107±3% (n = 6) for Dolt 4, 116±9% for Dorm 3 and 116±10% for Tort 2. For Se percent recovery of SRM were 100±5% (n = 6) for Dolt 4, 107±6% for Dorm 3 and 101±1% for Tort 2. Average Se and Hg concentrations from sample blanks were negligible (below detection for Se, and approximately <10% of sample Hg concentrations), thus blank corrections were not necessary. Dry weight sample detection limits for Hg and Se were 0.03 and 0.25 ppm, respectively, based on 3 standard deviations of the blank digests and an average sample digestion dilution of 500. Trace element concentrations were converted to a wet weight basis using dry weight to wet weight ratios measured for each sample.

### Data Analysis

Data were log-transformed to normalize and improve homogeneity of variance of individual variables. We tested for taxonomic patterns of Hg content, Se content and Hg:Se molar ratios with MANOVA, followed by Welch's ANOVA for individual variables in order to account for unequal variance among taxa. We examined Hg:Se ratios because Hg:Se molar ratios >1 are thought to indicate the presence of Hg in the body that is unbound to Se, potentially resulting in more toxic conditions than when Hg:Se values are <1 [49]. Taxonomic comparisons for all pairs were assessed using Tukey's HSD. We calculated coefficients of variation of Hg and Se content to compare interspecific and intraspecific variability between these two elements. We examined relationships between Hg or Se and Hg:Se ratios across taxa using linear regression on mean values for each taxa. The mean value for each taxon served as the unit for statistical analysis since individuals within taxa were not independent and to address issues associated with unequal sample sizes among taxa. Differences in slopes of these relationships between fish and invertebrates were tested with ANCOVA. Finally, we compared taxon-specific Hg concentrations to those from a Seafood Hg Database composed of Hg data for US commercial seafood items from multiple studies [12].

We found no significant differences in Hg concentrations for taxa collected across multiple seasons, sites or years, when such comparisons were possible. Therefore, we pooled data for the same taxa across sites and collection dates for all analyses. One exception was that Hg concentrations in softshell clams (*Mya arenaria*) were higher from Stony Brook Harbor than those from Smithtown Bay.

We conducted a Principal Components Analysis (PCA) to examine general relationships between Hg, Se food web and body size factors across taxa. We used mean values for each taxon (*sensu* [50], [51]), allowing us to address potential issues associated with nonindependence among individuals within taxa and unequal sample sizes. The PCA allows us to examine broad-scale relationships between food web and body size factors and Hg and Se content among taxa within the coastal ecosystem. Regional coastal food webs, specifically those along the north and south shores of Long Island, are qualitatively similar [52] and often linked through trophic interactions and regional migrations. We used stable isotopes that are commonly used to quantify relative food web position among taxa. These isotopes included δ^15^N, an indicator of trophic level [53] and lipid-normalized δ^13^C [54], indicating the relative importance of habitat-specific food sources in the diet, such as benthic versus pelagic food sources [53], [55], [56]. While this approach allows us to define relative position in the food web among taxa within the coastal ecosystem, we did not quantify specific feeding relationships that are highly dynamic and variable in natural systems. Total length was included as a single measure of body size instead of wet weight, because total length resulted in a PCA that explained a larger percentage of variation in the dataset than wet weight. Components with eigenvalues >1 were retained for interpretation [57], [58]. We also examined univariate relationships between 1) Hg concentration and δ^15^N, 2) Se concentration and δ^15^N, and 3) δ^13^C and δ^15^N across taxa.

## Results

### Taxonomic Patterns and Variability of Hg and Se

**Figure 1:**
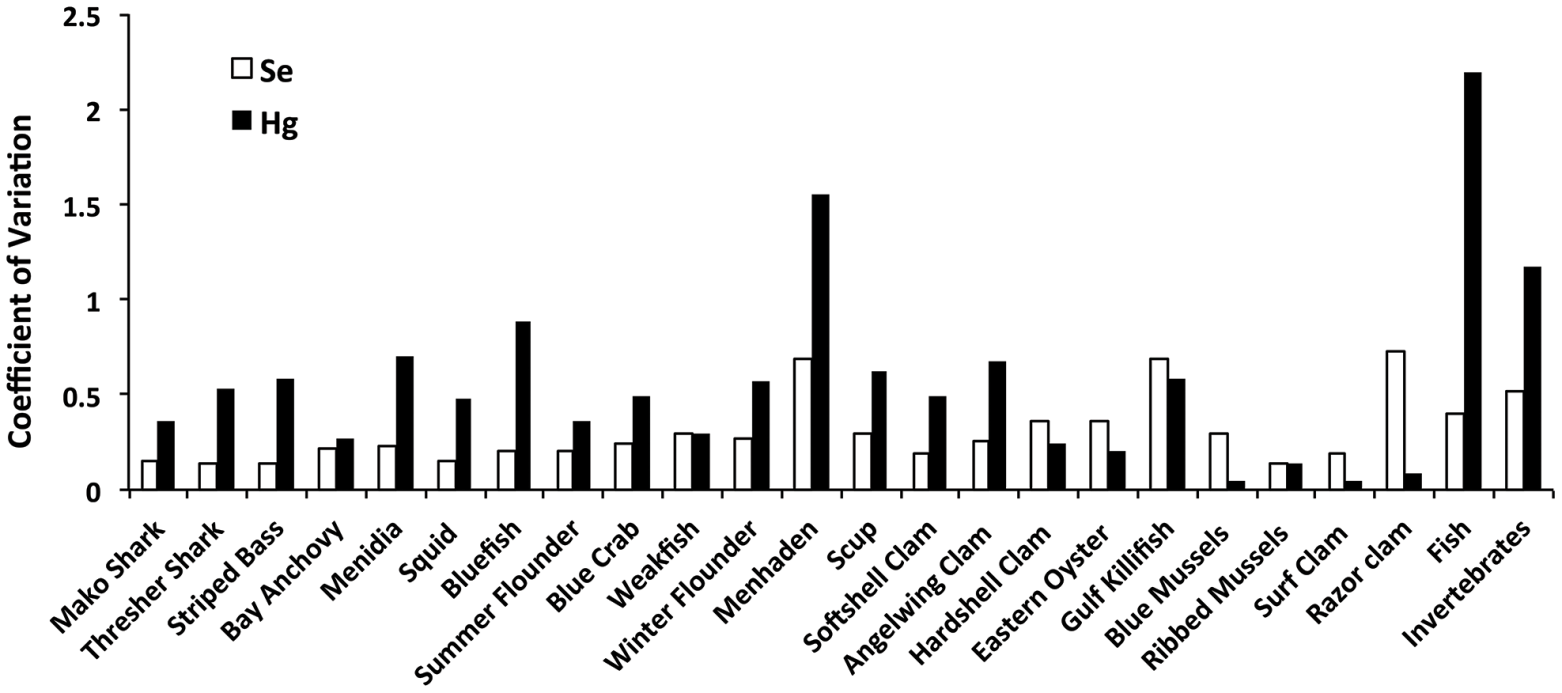
Variability in Hg and Se content in finfish and invertebrates. Hg content is more variable within and across taxa, compared with Se.

**Figure 2:**
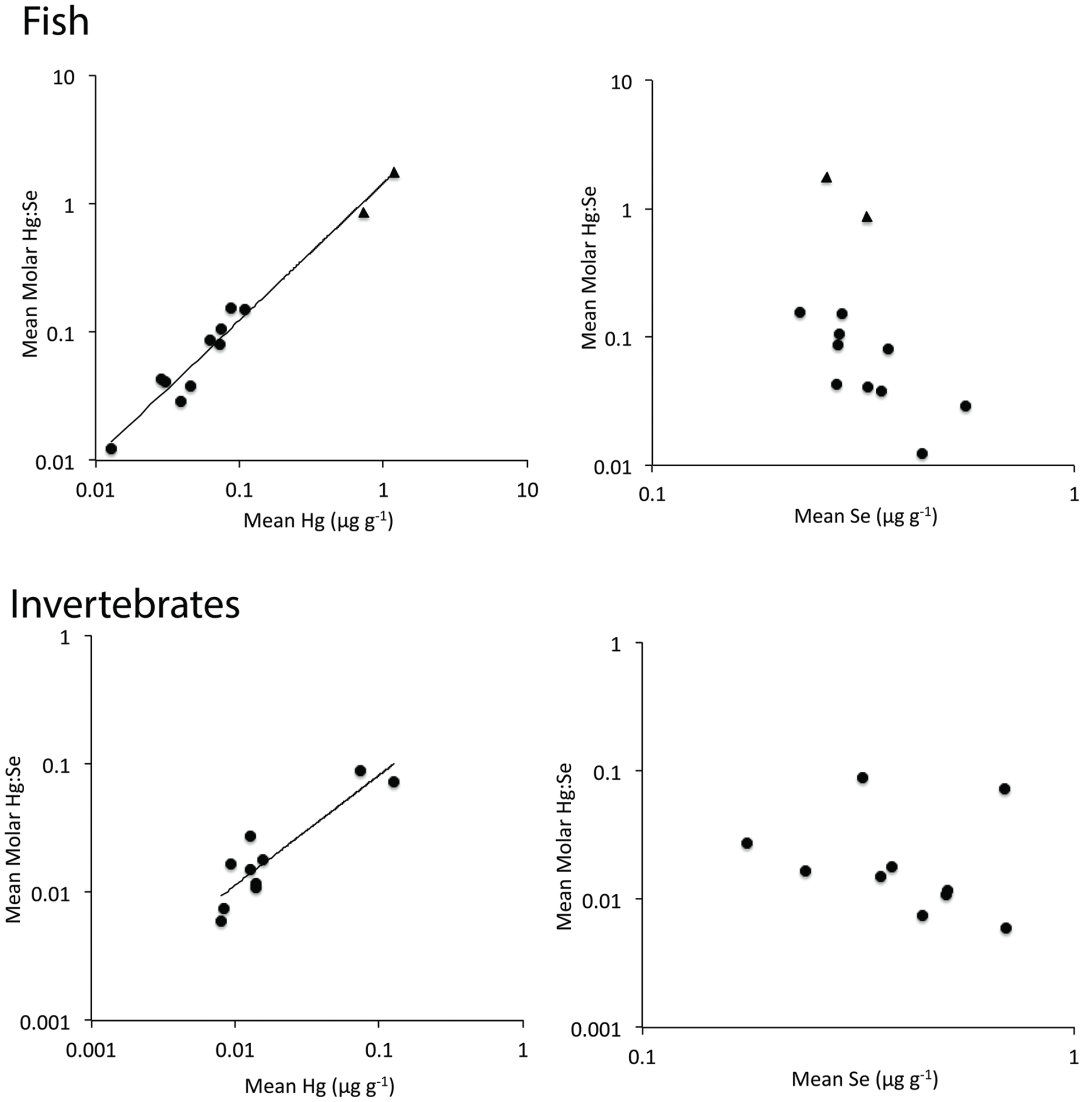
Relationships between Hg (left), Se (right) and Hg:Se molar ratios in fish (top) and invertebrates (bottom). Hg content is more strongly related to Hg:Se molar ratios than Se content. The range of the axes varies among panels. Shark taxa are shown as triangles. Hg and Hg:Se relationships are described by the equation for fish: Log_10_(Hg:Se)  = 0.17+1.0*Log_10_(Hg), R^2^ = 0.98, P<0.0001, F_1,10_ = 410 and invertebrates Log_10_(Hg:Se)  = −0.22+0.87*Log_10_(Hg), R^2^ = 0.78, P = 0.0006, F_1,8_ = 29.

We found clear taxonomic patterns in Hg, Se and Hg:Se molar ratios (Table 1). There were significant differences in Hg, Se and Hg:Se content among taxa (MANOVA, Pillai's Trace F_63, 534_ = 7.67, P<0.0001). In general, Hg was highest in shark, moderate in other finfish and lowest in bivalves. In contrast, Se was generally highest in bivalves and moderate to low in finfish and shark, depending on the species. Only mako shark had a Hg:Se ratio >1. Interspecific variability was higher in Hg than Se (Table 1), with the range of mean Hg content spanning three orders of magnitude, and mean Se content ranging within 1 order of magnitude among taxa. Additionally, intraspecific variability was higher for Hg than for Se, with a few exceptions for certain bivalve species and weakfish (Figure 1). Finally, Hg content explained 78% and 98% of the variability in mean Hg:Se across taxa for invertebrates and fish, respectively (Figure 2). When blue crab and squid (invertebrate taxa with the two highest mean Hg content) were removed from this analysis for invertebrates, the Hg-Hg:Se relationship was weak and nonsignificant (P = 0.23), indicating a strong influence of these two, non-bivalve invertebrate taxa. In contrast with Hg, Se content had no significant relationship with Hg:Se ratios for both fish and invertebrates. However, when blue crab and squid were removed from the analysis, the Se-Hg:Se relationship became negative and significant (F_1,6_ = 20.0, P = 0.004), and Se content explained 77% of the variation in molar Hg:Se. Similarly, when shark were excluded from the analysis, the Se-Hg:Se relationship for fish became marginally significant (F_1,8_ = 5.7, P = 0.04) and Se content explained 41% of the variation in molar Hg:Se. Although the slope of the Hg-Hg:Se relationship estimated for fish was greater than the slope for invertebrates, these differences in slope were not statistically significant (ANCOVA, F_1,1_ = 1.6, P = 0.22, for the interaction between taxonomic category and Hg).

**Table 1:** Taxonomic differences in Hg, Se and molar Hg:Se ratios (ppm, wet weight), shown in approximately decreasing values from top to bottom, according to statistically significant taxonomic differences (taxa not connected by the same letter(s)).

Taxon (N)^a^	Hg (Mean ±SE)	Taxonomic Differences^b^	Taxon (N)	Se (Mean ±SE)	Taxonomic Differences^c^	Taxon (N)	Hg:Se (Mean ±SE)	Taxonomic Differences^d^
Mako Shark (5)	1.184±0.192	A	Blue Crab (18)	0.689±0.041	A	Mako Shark (5)	1.761±0.203	A
Thresher Shark (5)	0.729±0.174	A	Razor clam (3)	0.694±0.293	ABC	Thresher Shark (5)	0.861±0.176	A
Blue Crab (18)	0.127±0.015	B	Scup (14)	0.553±0.044	AC	Bay Anchovy (9)	0.150±0.009	B
Bay Anchovy (9)	0.108±0.010	BC	Ribbed Mussel (3)	0.507±0.042	ABCD	Striped Bass (23)	0.154±0.020	B
Striped Bass (23)	0.086±0.010	BC	Blue Mussel (3)	0.508±0.086	ABCD	Atlantic silverside (15)	0.106±0.019	BC
Summer Flounder (11)	0.072±0.008	BCD	Surf Clam (2)	0.445±0.061	ABCDEF	Long-finned Squid (3)	0.088±0.022	BCDEF
Long-finned Squid (3)	0.074±0.020	BCDEF	Killifish (3)	0.436±0.172	ABCDEF	Summer Flounder (11)	0.080±0.008	BCE
Atlantic silverside (15)	0.074±0.013	BCDF	Angelwing Clam (3)	0.379±0.056	ABCDEF	Bluefish (10)	0.086±0.021	BCEF
Bluefish (10)	0.062±0.017	CDEF	Summer Flounder (11)	0.361±0.022	BDE	Blue Crab (18)	0.073±0.008	CEF
Scup (14)	0.039±0.006	DEFG	Eastern Oyster (4)	0.356±0.064	BCDEF	Weakfish (5)	0.043±0.005	CDEFG
Weakfish (5)	0.029±0.004	DEFGHI	Long-finned Squid (3)	0.324±0.028	BCDEFG	Winter Flounder (44)	0.040±0.004	DG
Winter Flounder (44)	0.031±0.003	EGH	Thresher Shark (5)	0.323±0.020	BCDEF	Menhaden (6)	0.038±0.012	DEFGH
Menhaden (6)	0.046±0.029	EFGHI	Winter Flounder (44)	0.324±0.013	DE	Softshell Clam (6)	0.028±0.004	DFGH
Blue Mussel (3)	0.014±0.0004	EGHI	Menhaden (6)	0.350±0.099	DEFG	Scup (14)	0.029±0.005	DGH
Ribbed Mussel (3)	0.014±0.001	EGHI	Bay Anchovy (9)	0.281±0.020	DEFG	Hardshell Clam (5)	0.017±0.003	GHI
Angelwing Clam (3)	0.015±0.006	EGHI	Atlantic silverside (15)	0.277±0.016	DEFG	Eastern Oyster (4)	0.015±0.002	GHI
Eastern Oyster (4)	0.013±0.001	GHI	Bluefish (10)	0.276±0.018	DEFG	Angelwing Clam (3)	0.018±0.008	GHI
Killifish (3)	0.013±0.004	GHI	Weakfish (5)	0.274±0.036	DEFG	Killifish (3)	0.012±0.002	GHI
Softshell Clam (6)	0.013±0.003	HI	Mako Shark (5)	0.259±0.018	DEFG	Blue Mussel (3)	0.012±0.002	GHI
Hardshell Clam (5)	0.009±0.001	I	Hardshell Clam (5)	0.238±0.038	EFG	Ribbed Mussel (3)	0.011±0.001	GHI
Surf Clam (2)	0.008±0.0003	GHI	Striped Bass (23)	0.224±0.007	FG	Surf Clam (2)	0.008±0.001	HI
Razor clam (3)	0.008±0.0004	HI	Softshell Clam (6)	0.175±0.013	G	Razor clam (3)	0.006±0.002	I

a Mako Shark, *Isurus oxyrinchus.*; Thresher Shark, *Alopias vulpinus*; Blue Crab, *Callinectes sapidus*; Striped Bass, *Morone saxatilis*; Summer Flounder, *Paralichthys dentatus*; Long-finned squid, *Loligo pealei*; Atlantic Silverside, *Menidia menidia*; Bluefish, *Pomatomus saltatrix*; Scup, *Stenotomus chrysops*; Weakfish, *Cynoscion regalis*; Winter Flounder, *Pseudopleuronectes americanus*; Menhaden, *Brevoortia tyrannus*; Blue Mussel, *Mytilus edulis*; Ribbed Mussel, *Geukensia demissa*; Angelwing Clam, *Cyrtopleura costata*; Eastern Oyster, *Crassostrea virginica*; Killifish, *Fundulus sp.*, Softshell Clam, *Mya arenaria*; Hardshell clam, *Mercenaria mercenaria*; Surf clam, *Spisula solidissima*; Razor Clam, *Ensis directus*.

b Welch ANOVA for Hg: F(21,29.6) = 77.31, P<0.0001;

c Welch ANOVA for Se: F(21,26.5) = 15.90, P<0.0001;

d Welch ANOVA for Hg:Se ratio: F(21,27.5) = 76.2, P<0.0001.

**Figure 3:**
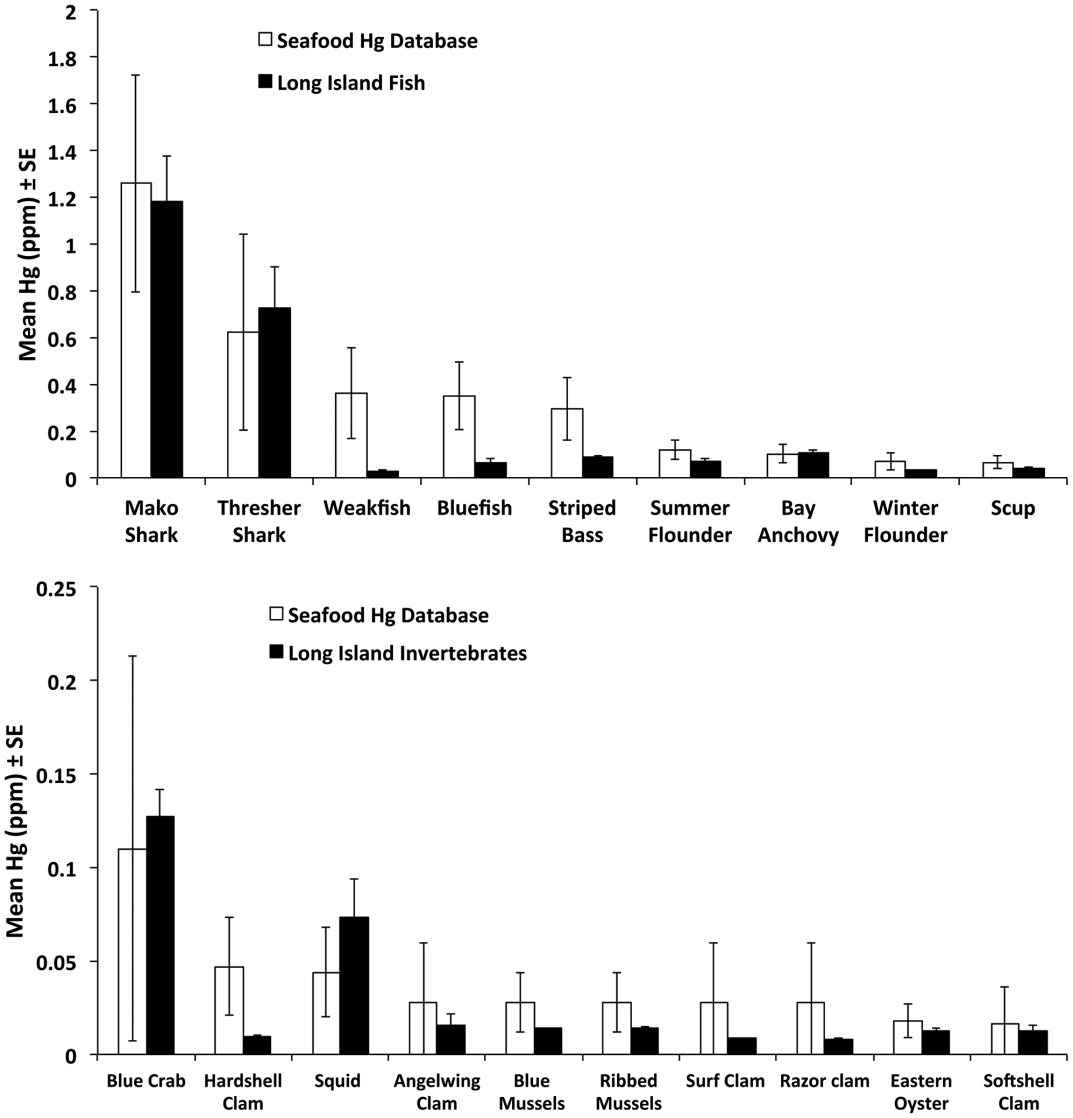
Mean mercury concentrations in Long Island fish (top) and invertebrates (bottom) were within the range, but generally lower than those summarized from a database of Hg concentrations in U.S. commercial seafood [Seafood Hg Database, 12]. Database taxa matched Long Island taxa except for bay anchovy (database values for all anchovies were used), long-finned squid (database values for all squid were used), angelwing clam, surf clam and razor clam (database values for all clams were used), ribbed mussels and blue mussels (database values for all mussels were used).

Finally, we found that mean mercury concentrations were lower in most invertebrate taxa (8 of 10) and finfish taxa (7 of 9) from our study compared with Hg concentrations in these taxa across other studies summarized in the Seafood Hg Database of US commercial seafood taxa [12] (Figure 3). Exceptions in which mean mercury concentrations were higher from our study include blue crab, squid, common thresher shark, and bay anchovies.

### Relationships between Hg, Se, Food Web Factors and Body Size

**Figure 4:**
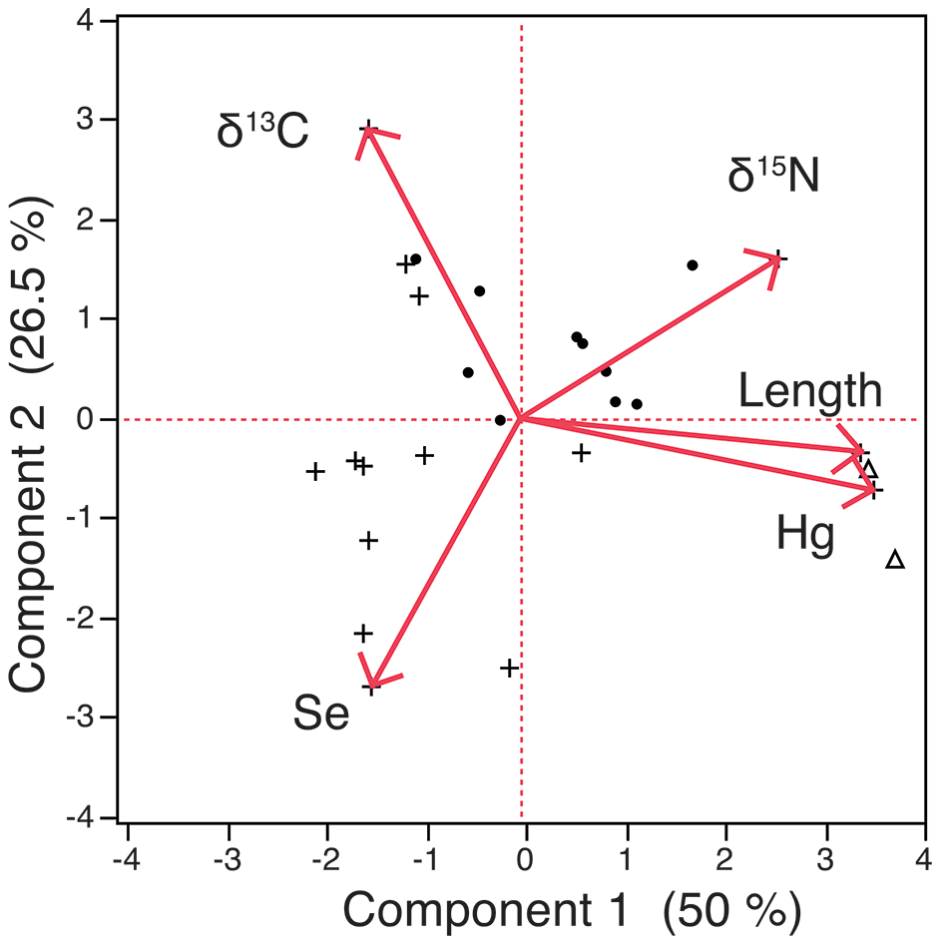
PCA biplot on mean values across taxa. Hg content is more strongly, positively related to body size and trophic level (δ^15^N). Invertebrates are indicated by a plus sign, shark species are indicated by a triangle, all other finfish are indicated by a circle.

The PCA showed clear, positive associations between Hg, body size (length) and trophic level (δ^15^N), consistent with bioaccumulation and biomagnification, respectively (Figure 4). These associations were reflected in Component 1, on which Hg, total length, and δ^15^N loaded strongly and positively (Table 2). We also found a positive relationship between Hg and δ^15^N, when other factors (body size, δ^13^C, Hg content) were excluded (Figure S1). In contrast with Hg, Se content had a weak, negative association with body size and trophic level in the PCA model. In addition, we found no significant relationship between Se and δ^15^N when the other factors were excluded (Figure S2). Both Hg and Se content were moderately, positively associated with feeding on pelagic food sources, whether through direct or indirect consumption, indicated by relatively low δ^13^C [41], [55], [56]. We found no significant relationship between δ^15^N and lipid-normalized δ^13^C (Figure S3). Additionally, the PCA showed clear separation between fish and invertebrates relative to Hg, Se, food web and body size factors. Together, the first two components explained 77% of the variation among observations in the dataset. Exclusion of both shark species, the largest sized and highest trophic level fish of our study, from the PCA yielded similar results with minimal reduction in the percent variance explained by the first two components (74%).

**Table 2:** Principal component eigenvalues, percent variance explained and variable loadings (loadings with an absolute value >0.4 in bold).

	*Component 1*	*Component 2*
Eigenvalue	2.50	1.32
% Variance	50.01	26.54
Cumulative%	50.01	76.54
Se	−0.39	**−0.71**
Hg	**0.94**	−0.19
Total Length	**0.91**	−0.09
δ^15^N	**0.69**	**0.43**
δ^13^C	**−0.41**	**0.77**

## Discussion

Our main finding, that Hg and Se concentrations exhibit contrasting patterns through the marine food web, has nutritional and toxicological implications for fish and shellfish consumers. In general, Hg concentrations increase at successively higher trophic levels in the marine food web while Se decreases. Thus, potential public health risks based on Hg and Se alone increases with trophic level. These findings, taken together with previous studies for non-commercial marine and freshwater taxa [15], [17], [19], indicate that differences in Hg and Se patterns, and their relationships with food web factors and body size, generally occur across ecosystems. Moreover, the simultaneous evaluation of Hg and Se in commercially important biota have not been commonly assessed and this study provides a basis for understanding Hg:Se relationships. Specifically, Hg concentrations, which ranged widely among and within taxa, tend to drive Hg:Se ratios, and are strongly related to food web factors and body size relative to Se. Our findings therefore help improve our understanding of the links between environmental factors, seafood quality and potential toxicity for piscivorous wildlife and humans.

Our study points to specific types of marine biota that are primary sources of Se or Hg for piscivorous wildlife and humans. Our finding that bivalves are high in Se is consistent with previous observations that bivalves are efficient Se accumulators [13], [14], and with conclusions that bivalves can be important sources of Se to higher trophic level wildlife and humans [24], [38]. This can explain, for example, our observation that scup, or porgy, which predominantly feed on bivalves [46], were higher in Se than other finfish. The higher Se content of bivalves is likely due to their relatively slow loss of assimilated Se, which may result from the reabsorption of Se-bound amino acids unique to bivalve digestion [14]. Finfish, particularly shark, have higher Hg concentrations than bivalves, consistent with previous observations [12], [16], and biomagnification of methylmercury in aquatic food chains. One implication of these findings is that for consumers of high trophic level, high Hg fish, any protection against Hg bioaccumulation or toxicity afforded through simultaneous exposure to Se from seafood is limited. Our findings also suggest that the major processes behind this pattern include strong bioaccumulation of Hg over time as body size increases, and relatively strong biomagnification of Hg through the food web compared with Se.

While the taxonomic Hg and Se patterns we found were similar to other studies, taxon-specific Hg concentrations from this study were, on average, slightly lower but within the range of values from the Seafood Hg Database of US commercial seafood samples from multiple geographic regions [59]. Hg and Se concentrations from this study, and many Hg concentrations within the Seafood Hg Database, were measured using ICPMS. Therefore, differences in analytical methods are not likely to explain this pattern. A previous study also found relatively low Hg content in mussels collected from the Long Island Sound compared to other sites throughout the US [60]. Differences in Hg inputs from the atmosphere likely do not explain lower Hg content of Long Island seafood species, because such inputs are relatively elevated in the Northeast US compared to other regions of the US [61]. Alternatively, differences in food web structure may strongly influence differences in Hg content among ecosystems [62]. Also, differences in mean Hg content may reflect differences in taxonomic identity between our study and the Seafood Hg Database. For example, we compared Hg content of one species of squid from our study (*Loligo pealei*) to a broader taxonomic group consisting of multiple squid species from the database. Overall, sedimentary production is the primary source of methylmercury to the Long Island Sound [63], and is likely to influence Hg content of Long Island marine fish and shellfish relative to other sites. One exception to the general pattern of relatively low Hg values in our study is that certain forage fish species were unexpectedly high in Hg given their known feeding habits and relatively low trophic level. For example, bay anchovies, which are plankton feeders, were relatively high in Hg, although within the range measured across anchovy species (mean  = 0.103, range  = 0.008-0.154) [12]. Another study also found that bay anchovies in Florida Bay are high in Hg (mean Hg  = 0.189, SD = 0.106) [64]. Atlantic silversides (*Menidia menidia*) were another species of forage fish that were relatively high in Hg, consistent with another study that found similar, high Hg in a congener species (Mississippi silverside) in San Francisco Bay [65].

The higher variability of Hg concentrations than of Se, as observed previously [16], largely accounts for the variability in molar Hg:Se patterns across taxa and may have implications for Hg-Se interactions and toxicity in seafood species. The high variability in Hg content likely explains the high variability in Hg:Se molar ratios observed within and among fish species [66]–[68]. Since Hg content in marine animals is primarily from dietary sources [25], [26], high Hg variability in animals likely reflects variability of Hg dietary exposure. Dietary exposure in natural populations is difficult to estimate. For example, sediment concentrations of these elements do not directly reflect exposure concentrations to fish. Nevertheless, sediment concentrations indicate that variability of these elements in the environment does not match the higher Hg variability in biota compared with Se. Specifically, sediment concentrations of total Hg (CV: 0.83) are only somewhat more variable than total Se (CV: 0.53) across sites in the Long Island Sound [60]. In addition, methylmercury, the chemical form that is efficiently transferred through the food web, is less variable than inorganic Hg in Long Island Sound sediments [69]. The higher variability of Hg may be partly due to the relative inability of organisms to maintain somatic concentrations of Hg over a range of exposure concentrations compared with Se [16], [70]. A few experimental studies have examined Hg and Se interactions, including the potential for Se to protect against Hg accumulation and toxicity [71]. However, such studies generally examine single exposure concentrations of Hg and Se. Additional studies are needed to examine responses in Hg and Se content to a range of both Hg and Se exposure concentrations simultaneously, while accounting for potential Hg-Se interactions [10].

Regardless of the underlying causes of higher Hg variability, the comparatively narrow range of Se concentrations in teleosts that we found may result in toxic conditions if methylmercury concentrations are high, either because the latter is not bound by free selenium or because the mercury reduces available selenium concentrations to sub-optimal levels [9], [49]. Thus, the protective effect of Se may be particularly limited for larger, higher trophic level organisms because they are more likely to have relatively high Hg content [72]. However, Se deficiency due to Hg-Se binding is more likely for organisms with molar Hg:Se ratios ∼1 [9], observed only for thresher shark in our study. Finally, because the efflux rates of methylmercury (loss of assimilated methylmercury from the body) are much lower than those of Se [31], except for bivalves, differences in tissue concentrations of assimilated Hg and Se may increase in fish over time after exposure. In marine environments with relatively minor Se inputs, such as the Long Island Sound, Se will likely play a minor protective role with respect to Hg accumulation. The same may not be true in systems that receive both high Se and Hg inputs [73].

In general, Hg concentrations were more strongly linked with food web and body size factors than Se concentrations, reflecting clear differences in bioaccumulation and trophic transfer processes between these two elements. First, our finding that increasing length across taxa is strongly associated with higher Hg content, in contrast with slight decreases in Se content, reflects taxonomic differences in Hg and Se content (i.e., higher Se concentrations in smaller-sized invertebrates than in fish). These intraspecific body size relationships are analogous to interspecific body size relationships in which Hg content strongly increases, while Se content nonlinearly decreases with body size [74]. Both intra and interspecific body size relationships are consistent with generally lower efflux rates of methylmercury than Se, because lower efflux rates lead to greater bioaccumulation over time as body size increases. Second, we found that Hg clearly biomagnifies strongly relative to Se when these elements are compared among taxa in the same ecosystem. While Se was generally lower in higher trophic level organisms, we also found that scup have slightly higher Se content than most bivalves, its predominant prey [46]. Thus, exceptions to the broad pattern of decreasing Se concentrations up the food chain emerge when examining specific feeding relationships that are not captured by stable isotope analysis. Stronger biomagnification of Hg relative to Se likely continues up the food chain, leading to total Hg concentrations exceeding Se concentrations in muscle tissue of waterfowl [75] and other piscivores, including humans. Finally, pelagic feeding (lower δ^13^C values) was moderately associated with higher content of both Hg and Se. At least two other studies found positive associations between pelagic feeding and Hg content [41], [42]. Our results suggest that overall, consuming a pelagic-based diet has less influence on Hg and Se content compared with body size and trophic level effects.

While relationships between Hg concentration, trophic level and body size are established for fish from the Long Island Sound and elsewhere [34], [36], [76], [77], relationships between Hg and other nutritional and toxicological factors such as Se are relatively rare in the literature. Hg-Se comparisons are necessary to understand potential interactions between these two elements and to inform efforts to incorporate Se into Hg risk assessment [11]. There is now a need to reconcile our observations of Hg-Se interactions at the molecular [9] and organism level [10], [71] from controlled, experimental studies, with known differences in Hg and Se bioaccumulation patterns within the food web. More generally, future comparative studies should include other nutritional (e.g., omega-3 fatty acids) and toxicological factors (e.g., PCBs or other persistent organic pollutants), in order to understand the overall dietary quality of marine taxa, and to predict how such factors may co-vary with ecological changes.

## Supporting Information

Figure S1 **Relationship between Hg content and trophic level (δ^15^N).** Invertebrates are indicated by a plus sign, shark species are indicated by a triangle, all other finfish are indicated by a circle. (R^2^ = 0.27, F_1,20_ = 7.52, P = 0.01).
(PDF)

Figure S2 **No significant relationship between Se content and trophic level (δ^15^N).** Invertebrates are indicated by a plus sign, shark species are indicated by a triangle, all other finfish are indicated by a circle. (P = 0.10).
(PDF)

Figure S3 **No significant relationship between habitat-specific feeding (δ^13^C) and trophic level (δ^15^N).** Invertebrates are indicated by a plus sign, shark species are indicated by a triangle, all other finfish are indicated by a circle. (P = 0.90).
(PDF)
