# Correction: Contrasting Food Web Factor and Body Size Relationships with Hg and Se Concentrations in Marine Biota

**DOI:** 10.1371/annotation/3a79d01e-e0e3-4636-8416-167aaa6df3e5

**Published:** 2013-11-05

**Authors:** Roxanne Karimi, Michael Frisk, Nicholas S. Fisher

There was an error in the name of the second author.

The correct name of this author is: Michael G. Frisk

The correct citation is: Karimi R, Frisk MG, Fisher NS (2013) Contrasting Food Web Factor and Body Size Relationships with Hg and Se Concentrations in Marine Biota. PLoS ONE 8(9): e74695. doi:10.1371/journal.pone.0074695

The correct version of the Author Contributions section is: Conceived and designed the experiments: RK MGF NSF. Performed the experiments: RK. Analyzed the data: RK MGF. Contributed reagents/materials/analysis tools: MGF NSF. Wrote the paper: RK MGF NSF. 

