# Supplementary figures and images for: Contrasting Food Web Factor and Body Size Relationships with Hg and Se Concentrations in Marine Biota
Source: PLoS One. 2013 Sep 3;8(9):e74695. doi: 10.1371/journal.pone.0074695 (PMC3760827; doi:10.1371/journal.pone.0074695)

Figure S1.

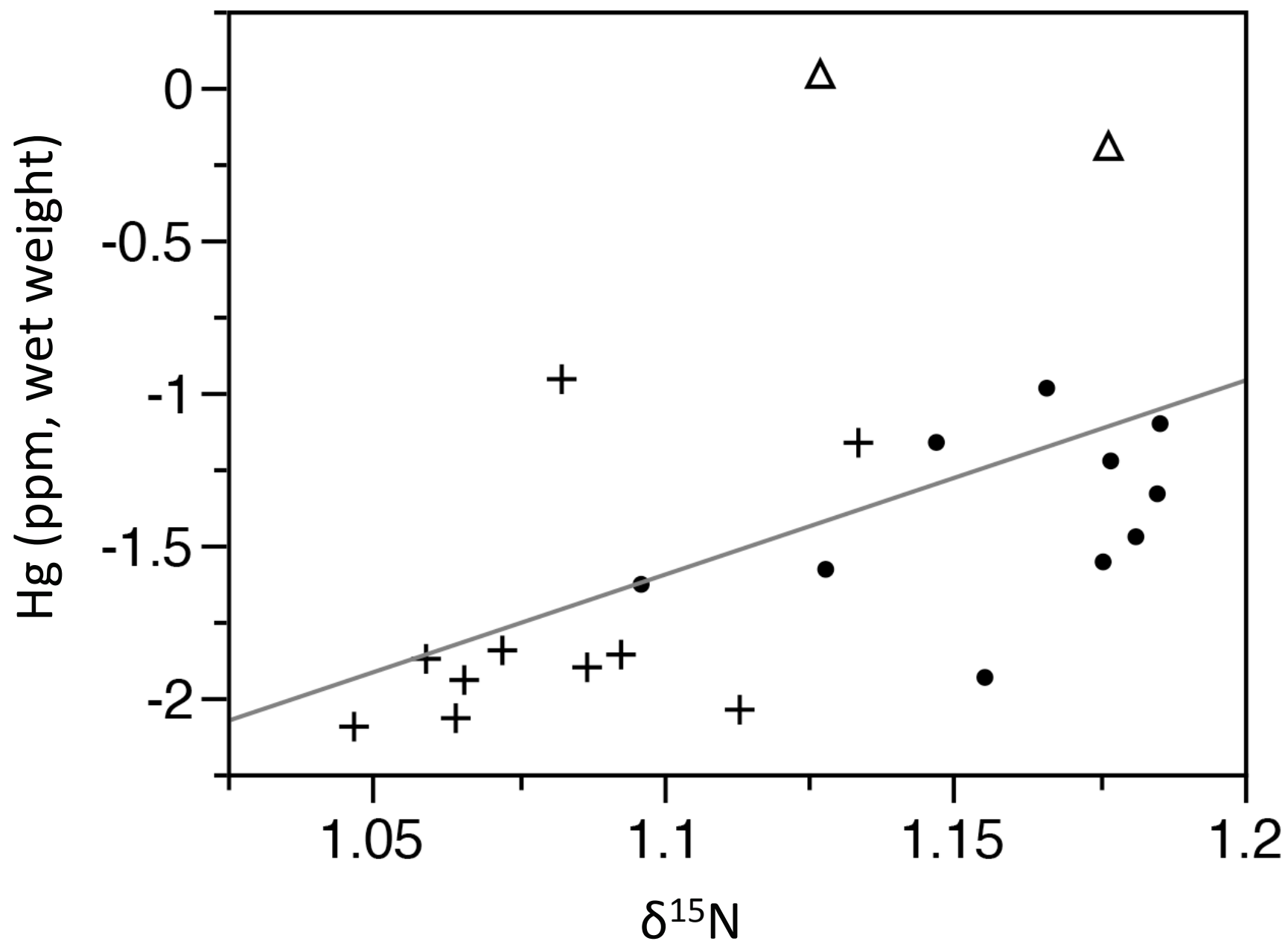

Supplement: Figure S1 — Relationship between Hg content and trophic level (δ15N). Invertebrates are indicated by a plus sign, shark species are indicated by a triangle, all other finfish are indicated by a circle. (R2 = 0.27, F1,20 = 7.52, P = 0.01). (PDF) [file pone.0074695.s001.pdf]

Figure S2.

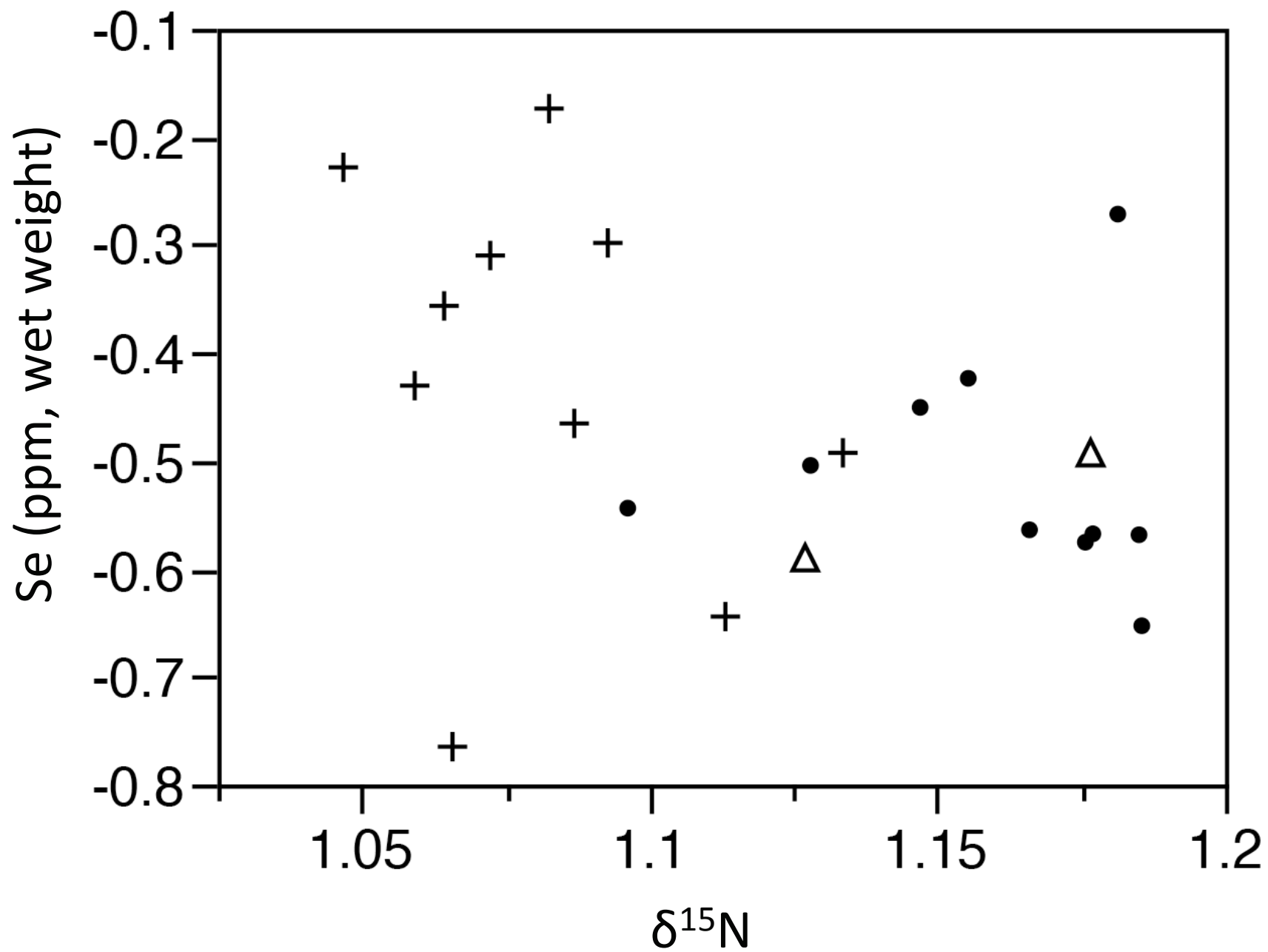

Supplement: Figure S2 — No significant relationship between Se content and trophic level (δ15N). Invertebrates are indicated by a plus sign, shark species are indicated by a triangle, all other finfish are indicated by a circle. (P = 0.10). (PDF) [file pone.0074695.s002.pdf]

Figure S3.

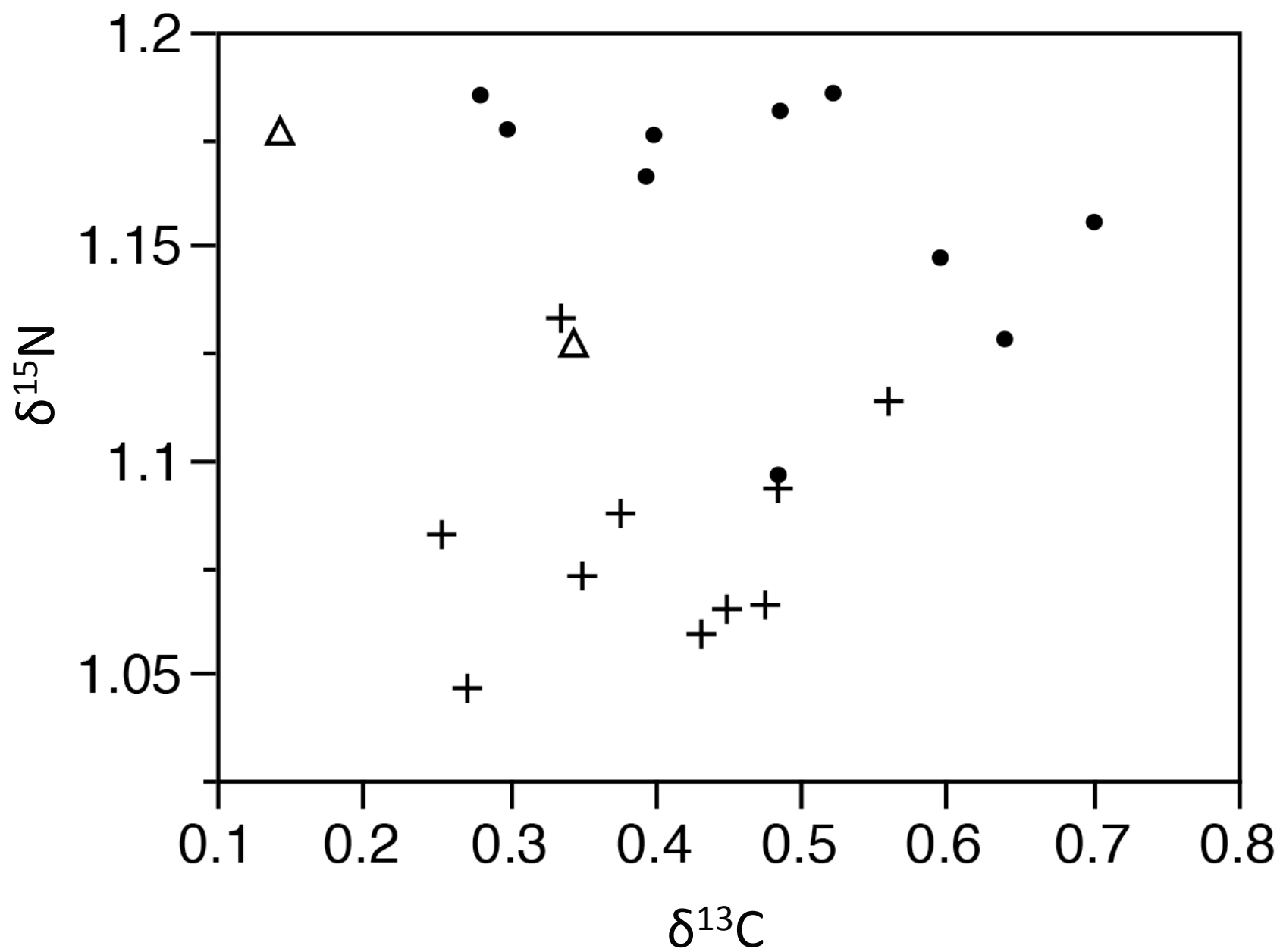

Supplement: Figure S3 — No significant relationship between habitat-specific feeding (δ13C) and trophic level (δ15N). Invertebrates are indicated by a plus sign, shark species are indicated by a triangle, all other finfish are indicated by a circle. (P = 0.90). (PDF) [file pone.0074695.s003.pdf]
